# Establishment and Characterization of a Stable hERG Cell Line for High-Throughput Drug Cardiac Safety Screening

**DOI:** 10.3390/ijms27083701

**Published:** 2026-04-21

**Authors:** Hailin Lu, Qingqing Guo, Qinling Qiu, Jiying Hu

**Affiliations:** 1 High-Throughput Screening Center, Shenzhen Bay Laboratory, Shenzhen 518132, China; luhl@szbl.ac.cn; 2 Biomedical Research Core Facility, Shenzhen Bay Laboratory, Shenzhen 518132, China; guoqq@szbl.ac.cn; 3 Institute of Molecular Physiology, Shenzhen Bay Laboratory, Shenzhen 518132, China; qqiuaf@connect.ust.hk

**Keywords:** hERG channel, stable cell line, automated patch-clamp, drug safety screening

## Abstract

The hERG potassium channel is critical for cardiac ventricular repolarization and a core target in pre-clinical drug safety screening. A robust, stable cell line with uniform, high hERG expression is essential for high-throughput assessments. In this study, we established a functional stable HEK293T cell line with high hERG expression. The hERG gene was subcloned into Lenti-HA-hERG-P2A-EGFP plasmid, in which GFP serves as a selection marker via a P2A self-cleaving peptide. GFP-positive monoclonal cells were isolated by fluorescence-activated cell sorting (FACS). Confocal imaging confirmed that hERG localized predominantly to the cell membrane, consistent with its physiological role. Manual patch-clamp revealed canonical hERG current properties: a small, stable current during depolarization to 20 mV, followed by a large outward tail current upon repolarization to −40 mV-a hallmark of hERG channel gating. Automated patch-clamp (APC)-based current profiling showed 93.5% of stable hERG cells exhibited peak tail currents > 50 pA (87% > 100 pA, with 49.5% > 400 pA), whereas 100% of blank HEK293T cells showed peak tail currents < 50 pA. Pharmacological validation with E-4031 demonstrated concentration-dependent inhibition of hERG currents, with an IC_50_ of 29.8 nM, which is consistent with literature-reported values. The stable hERG-expressing HEK293T cell line developed here exhibits consistent hERG expression, canonical channel function, and physiological sensitivity to hERG blockers. When paired with high-throughput APC systems, this cell model provides a robust, standardized platform for pre-clinical drug-induced hERG inhibition evaluation, aiding early detection of long QT syndrome risks and safer drug development.

## 1. Introduction

Cardiovascular safety assessment is a pivotal milestone in pre-clinical drug development, as drug-induced cardiovascular toxicities remain a leading cause of late-stage clinical trial failures and post-marketing withdrawals [1]. To standardize global cardiovascular safety testing, the International Council for Harmonisation of Technical Requirements for Pharmaceuticals for Human Use (ICH) established the ICH S7B guideline, which mandates evaluating drug candidates for arrhythmia risk—particularly risk linked to electrocardiogram (ECG) QT interval prolongation [2]. The QT interval reflects ventricular depolarization and repolarization duration; its prolongation is a well-validated biomarker for increased risk of torsades de pointes (TdP), a life-threatening polymorphic ventricular arrhythmia [2].

The hERG potassium channel, encoded by the human Ether-à-go-go Related Gene (hERG), is the primary mediator of cardiac ventricular repolarization, facilitating outward potassium currents (IKr) that restore the myocardial electrical gradient after each heartbeat [3]. Drug-induced hERG channel block delays repolarization, directly prolonging the QT interval and increasing drug-induced long QT syndrome risk [3]. ICH S7B strongly recommends hERG-expressing cell-based ion channel assays as a core component of nonclinical safety testing [2]; these assays enable direct measurement of drug-induced hERG inhibition, allowing researchers to prioritize compounds with low cardiotoxicity risk early in development and thereby reducing late-stage failures and improving patient safety [2].

Traditionally, manual patch-clamp has been the “gold standard” for hERG current measurement. This technique uses glass microelectrodes to quantify electrical activity of hERG channels in either induced hERG-expressing cells or native cardiomyocytes, offering high precision for channel gating kinetics and drug sensitivity analyses [4]. However, its reliance on specialized expertise, low throughput of just about 5 compounds/day per operator and labor-intensive workflow make it impractical for screening the thousands of candidates typical of modern drug discovery [4].

Alternative high-throughput methods, including radioligand binding assays and thallium flux assays, have been proposed to address this limitation. Radioligand assays quantify the competitive binding of test compounds to tritium-labeled dofetilide, a high-affinity hERG blocker, while thallium flux assays use thallium-based dyes to indicate intracellular potassium concentration changes [5,6]. Despite higher throughput, these methods are indirect, prone to false positives and negatives, and unable to quantify functional channel inhibition. Radioligand assays also pose safety and waste disposal challenges due to radioactive reagents, further limiting utility [5].

High-throughput automated patch-clamp (APC) systems (e.g., SyncroPatch 384i) have emerged as a transformative solution, combining patch-clamp accuracy with automation to enable screening of thousands of compounds daily [7]. APC uses planar chips to capture cells and form high-resistance seals, with robotic liquid handling streamlining solution exchange and data acquisition, thereby reducing human error and improving assay standardization [7]. A critical limitation of APC, however, is its reliance on random cell sampling, in contrast to manual patch-clamp which enables the selection of viable, high-expression cells. This requires cell models with uniform, high-level hERG expressions to ensure sufficient valid data [7].

Transient hERG transfection has been used to produce APC-compatible hERG-expressing cells but exhibits variable expression with only 70–80% of cells positive, reduced post-freeze–thaw viability, and batch-to-batch variability [8]. In contrast, stable cell lines ensure consistent, reliable hERG expression across passages and experiments, which is critical for generating reproducible and comparable data. Additionally, stable cell lines eliminate repeated transfection and cryopreservation needs, streamlining workflows and reducing variability from these steps [8].

Here, we report the construction and characterization of a stable HEK293T (human renal epithelial) cell line with high, uniform hERG expression. We validated its functionality using manual patch-clamp and high-throughput APC system, confirmed its sensitivity to the hERG-specific blocker E-4031 hydrochloride, and demonstrated its suitability for high-throughput drug safety screening. This model addresses key limitations of existing systems, providing a reliable platform for early-stage hERG-related cardiac safety assessment.

## 2. Results

**Figure 1:**
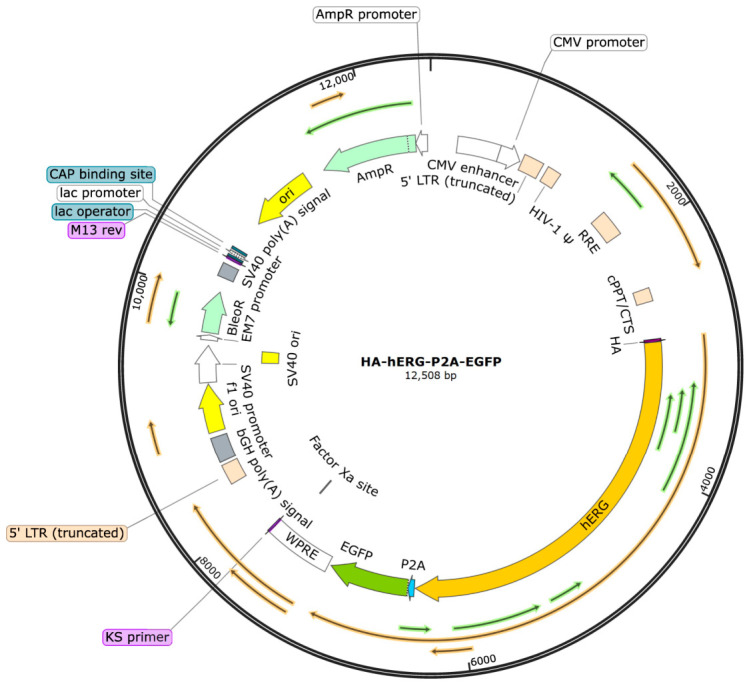
Schematic map of the Lenti-HA-hERG-P2A-EGFP plasmid. The plasmid is designed to co-express HA-tagged hERG and GFP, with the P2A self-cleaving peptide enabling the separation of the two proteins after translation.

The map of the hERG stable transfection plasmid is shown in Figure 1. Briefly, the hERG gene was cloned into the lentiCas9-EGFP vector and replaced the Cas9 sequence. An HA tag was fused to the N-terminus of the hERG gene, resulting in the construction of the Lenti-HA-hERG-P2A-EGFP plasmid. The GFP and hERG gene fragments are separated by a P2A self-cleaving peptide, which enables the co-expression of hERG and GFP. Notably, the finally expressed hERG protein does not carry the GFP tag.

**Figure 2:**
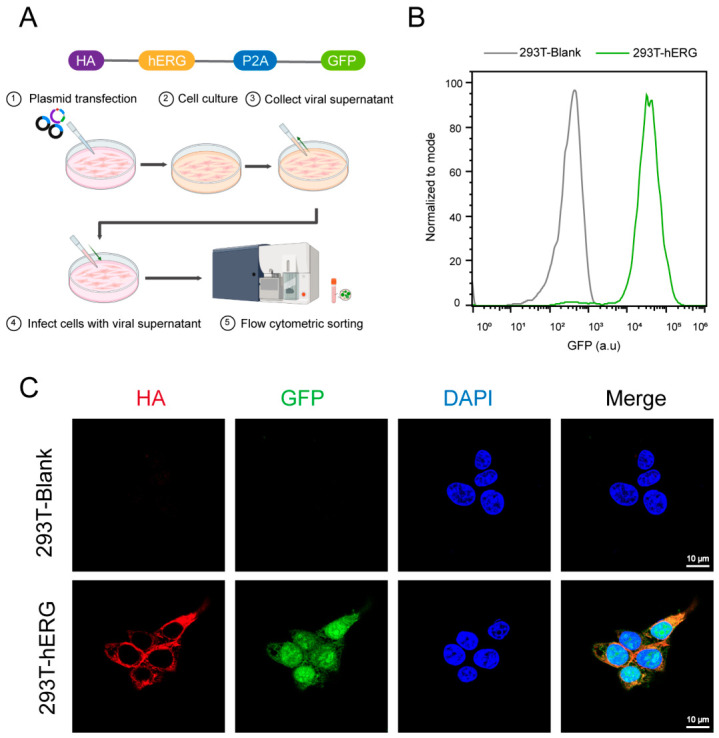
Generation of HEK293T-hERG stable cell line. (**A**) Schematic illustration of the workflow for generating HEK293T-hERG stable cell line, including plasmid transfection, cell culture, viral supernatant collection, cell infection, and flow cytometric sorting. The plasmid is designed to co-express HA-tagged hERG and GFP, with the P2A self-cleaving peptide enabling the separation of the two proteins after translation. (**B**) Flow cytometry analysis of GFP expression in 293T-blank and 293T-hERG cells. The green peak represents HEK293T-hERG cells with high GFP expression, while the gray peak represents 293T-blank cells with negligible GFP expression. (**C**) Confocal microscopy images showing the expression and localization of HA (red, labeling hERG) and GFP (green) in blank and hERG cells. DAPI (blue) stains the nuclei. Scale bar: 10 μm.

The cell line construction process is shown in Figure 2A. To construct a stable hERG cell line using lentivirus, a plasmid containing HA-tagged hERG and GFP linked by P2A was used (Figure 2A). The hERG cell strain was obtained by lentiviral infection, and GFP-positive cells were sorted by flow cytometry to obtain the hERG monoclonal cell strain. The flow cytometry sorting results are shown in Figure 2B. hERG cells showed a strong GFP signal, while blank cells transfected with the empty vector had no GFP expression. Next, we used confocal microscopy to observe the expression and cellular localization of hERG in the hERG cell strain obtained by flow sorting. As shown in Figure 2C, hERG-expressing cells exhibited clear HA (red, hERG marker) and GFP (green) signals, while blank 293T cells transfected with empty vector showed no HA and GFP expression. Merged images showed the majority of HA signals were distributed at the cell membrane, indicating the correct subcellular localization of hERG.

**Figure 3:**
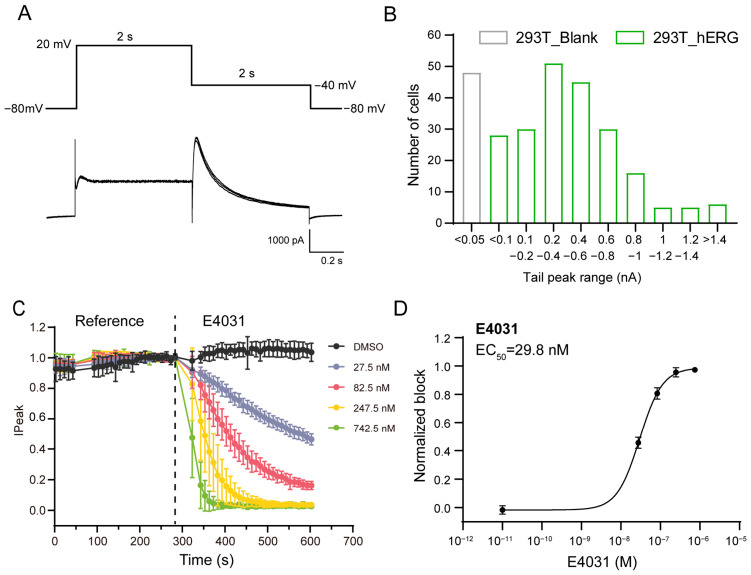
Functional characterization of hERG channels in HEK293T cells. (**A**) Representative current traces of hERG channels in stable hERG cells recorded via manual patch-clamp. The upper part shows the voltage protocol. The lower part displays the corresponding hERG currents, which are the superimposed traces from four independent biological replicates. The scale bar indicates 1000 pA for current and 0.2 s for time. (**B**) Distribution of peak tail currents in 293T-blank and 293T-hERG cells detected by APC. The histogram illustrates the number of cells within different peak tail current ranges (nA), where blank cells (gray bars) mainly have peak tail currents less than 0.05 nA, while hERG cells (green bars) exhibit a wide range of larger peak tail currents. (**C**) Time-course of peak tail current inhibition by different concentrations of E-4031 (27.5 nM, 82.5 nM, 247.5 nM, 742.5 nM) and DMSO control. Replicate wells for each concentration: *n* = 27 (0 nM), *n* = 52 (27.5 nM), *n* = 46 (82.5 nM), *n* = 44 (247.5 nM), *n* = 47 (742.5 nM). All data were recorded by SyncroPatch 384i APC platform. (**D**) Concentration–response curve for E-4031-mediated inhibition of hERG channels, with the half-maximal inhibitory concentration (IC_50_) calculated to be 29.8 nM. Error bars represent standard deviation. All data were recorded using SyncroPatch 384i APC platform.

To verify the function of the hERG cell line using manual patch-clamp, voltage steps were applied. The membrane potential was first held at −80 mV, then stepped to 20 mV for 2 s, followed by a step to −40 mV for 2 s, and finally returned to −80 mV (Figure 3A). The resulting current traces showed typical hERG channel currents. Upon the depolarizing step to 20 mV, a small and relatively stable current was observed. When the membrane potential was stepped to −40 mV, a large outward tail current, characteristic of hERG channels, was elicited, which then decayed over time (Figure 3A). These current patterns are consistent with the known functional properties of hERG channels, indicating that the hERG cell line exhibits normal channel function. Next, the current traces and peak tail current distribution were detected by the APC system SyncroPatch 384i. Representative hERG channel current traces recorded from the stable cell line via APC are shown in Figure S1A (Supplementary Materials). As shown in Figure S1A, consistent with the canonical biophysical properties of hERG channels, the stable HEK293T-hERG cells exhibited rapidly activating and inactivating inward rectifier-like currents upon depolarization, with prominent tail currents observed during repolarization. Figure S1B shows a whole-plate screenshot of the APC experiments, with online analysis screenshots showing the changes in peak tail currents before and after E-4031 application. It can clearly be observed that the peak tail current amplitude differs substantially between hERG cells and blank cells; as indicated in Figure S1B, hERG-expressing cells were sensitive to the blocker, while blank cells showed no response.

As shown in Figure 3B and Table 1: 100% of blank 293T cells (*n* = 48) had currents < 50 pA; of 216 hERG cells tested, 93.5% (*n* = 202) had currents > 50 pA (87% > 100 pA), with 13.9% (*n* = 30) at 100–200 pA, 23.6% (*n* = 51) at 200–400 pA, 20.8% (*n* = 45) at 400–600 pA, and 28.7% (*n* = 62) > 600 pA.

The sensitivity of the hERG cell to the hERG-specific blocker E-4031 was detected using the SyncroPatch 384i APC system. hERG cells were treated with E-4031 (27.5, 82.5, 247.5, 742.5 nM) and DMSO (control); peak tail currents were recorded over time. As shown in Figure 3C, E-4031 induced concentration-dependent current reduction, confirming dose-dependent hERG block. Figure 3D shows the normalized E-4031 concentration–response curve. % block was calculated as [(control − drug)/control] × 100. Half-maximal inhibitory concentration (IC_50_) was calculated to be 29.8 nM, which is consistent with values from the literature [9], validating the cell line’s physiological relevance.

## 3. Discussion

In this study, we successfully established a stable HEK293T cell line with high hERG expression and canonical channel function, ideal for APC-based high-throughput screening. Lenti-HA-hERG-P2A-EGFP plasmid construction, lentiviral infection, and flow cytometric sorting ensured efficient, specific hERG expression—confirmed by confocal microscopy. Functional validation via manual patch-clamp and APC system demonstrated typical hERG currents. Manual patch-clamp revealed characteristic voltage-dependent current patterns, while SyncroPatch 384i showed that 87% of hERG cells exhibited peak tail currents greater than 100 pA, indicating robust function. In contrast, peak tail currents were below 50 pA in all blank control cells. Pharmacological testing with E-4031 yielded a concentration-dependent block and IC_50_ of 29.8 nM, which is consistent with previously reported values in the literature [9], confirming the cell line’s reliability for hERG inhibition assays.

Several hERG-expressing cell lines are currently commercially available; however, most commercial cell lines are associated with extremely high purchasing costs and often involve restrictive usage terms, which substantially limit their widespread application in basic research and early-stage drug screening, especially in resource-limited laboratories. This study provides a detailed, cost-effective, and technically feasible protocol for generating a functional hERG stable cell line with robust and reproducible channel activity validated by both manual and high throughput APC system. This practical construction strategy, together with the comprehensive functional characterization, represents meaningful incremental novelty for researchers seeking an affordable and reliable alternative tool for cardiac safety assessment.

## 4. Materials and Methods

Reagents and materials: Dulbecco’s Modified Eagle’s Medium (DMEM) (Gibco, C11995500BT), fetal bovine serum (FBS) (Gibco, 10099-141C), and penicillin–streptomycin (Gibco, 15140122) were purchased from Thermo Fisher, Waltham, MA, USA. The primary antibodies against HA (Cell Signaling Technology, Danvers, MA, USA, 3724) and β-tubulin (Abcam, Cambridge, UK, ab179513) were obtained commercially. The secondary antibody for immunofluorescence, the goat anti-mouse IgG (HA1006) was purchased from HUABIO, Hangzhou, Zhejiang, China. E-4031 (HY-15551) was purchased from MedChemExpress (MCE), Monmouth Junction, NJ, USA. The 293T cells used to establish the stable hERG-expressing cell line were purchased from ATCC (CRL-3216, Manassas, VA, USA).

Generation of stable HEK293T-hERG cell line: The stable HEK293T cell line overexpressing HA-tagged hERG was generated using a lentiviral delivery system. The hERG gene was cloned into the lentiCas9-EGFP vector, with an HA tag fused to its N-terminus to generate the Lenti-HA-hERG-P2A-EGFP plasmid. Specifically, the Cas9 fragment was excised via XbaI and BamHI double digestion, and the HA-hERG insert, with compatible cohesive ends generated by the same restriction enzymes, was directionally cloned into the linearized vector backbone to generate the Lenti-HA-hERG-P2A-EGFP plasmid. To confirm the fidelity of the cloning process, Sanger sequencing—performed by General Biosystems (Chuzhou, Anhui, China) Co., Ltd.—was conducted across the entire HA-hERG coding region, as well as the vector-insert junctions. The sequencing reads were aligned against the reference hERG sequence using DNAMAN version 9.0 software (Lynnon Biosoft, San Ramon, CA, USA). HEK293T cells were cultured in DMEM supplemented with 10% (*v*/*v*) FBS, 100 U/mL penicillin, and 100 U/mL streptomycin at 37 °C in 5% CO_2_. Replication-deficient lentivirus was produced by transiently transfecting 0.75 μg psPAX2, 0.25 μg pMD2.G, and 1 μg Lenti-HA-hERG-P2A-EGFP into HEK293T cells seeded in 6-well plates. Viral supernatants were collected at 48 h, filtered through a 0.45 μm filter, and diluted in fresh DMEM containing 10 μg/mL polybrene for HEK293T cell infection. At 48 h post-infection, cells were resuspended in fresh medium, and GFP-positive cells were sorted using CytoFLEX SRT (Beckman Coulter, Indianapolis, IN, USA).

Cell immunofluorescence: HEK293T cells were seeded onto four-chamber confocal culture dishes (J40204) and incubated for 24 h at 37 °C. Afterward, the cells were washed twice with PBS and fixed in freshly prepared 4% paraformaldehyde (PFA) for 15 min at room temperature. Following two washes with PBS, the cells were permeabilized and blocked with a blocking buffer (1 × PBS, 5% BSA, 0.3% Triton X-100) for 1 h at room temperature. The primary and secondary antibodies were diluted in the dilution buffer (1 × PBS, 1% BSA, 0.3% Triton X-100) according to the manufacturers’ recommendations. Cells were incubated with HA tag antibody for 1 h at room temperature. Subsequently, the cells were rinsed three times with PBS, followed by incubation with goat anti-mouse IgG antibody at room temperature in the dark for 1 h. After the cells were washed three times with PBS, DAPI was added to stain the nuclei according to the manufacturer’s instructions. Imaging was captured using an LSM980 laser scanning confocal microscope (Carl Zeiss Microscopy GmbH, Jena, Germany), and further image processing was performed using Image J 1.54r (National Institutes of Health, Bethesda, MD, USA).

Conventional patch-clamp recording: Whole-cell manual patch-clamp (HEKA Elektronik, Lambrecht, Germany) was used to verify hERG channel function. Glass pipettes (Biomedical Instruments, Zollnitz, Germany) were pulled with a DMZ puller (Zeitz Instruments, Martinsried, Germany) to 0.9–2.5 MΩ and filled with intracellular solution (ICS: 140 mM KCl, 1 mM EGTA, 10 mM HEPES, 10 mM NaCl, 18 mM sucrose; pH 7.33 with KOH, osmolarity 310 mOsm with sucrose). Cells were pre-incubated in extracellular solution (ECS: 140 mM NaCl, 3 mM KCl, 1 mM CaCl_2_, 1 mM MgCl_2_, 10 mM HEPES, 20 mM glucose, 0.2% DMSO; pH 7.4 with NaOH, osmolarity 305 mOsm with glucose) for ≥5 min. Leak current was subtracted online via a P/4 protocol; signals were sampled at 100 kHz and filtered at 10 kHz. After establishing whole-cell configuration (80% series resistance compensation, capacitive transient cancelation), an initial step protocol (depolarizing pulse every 10 s for 5 min) stabilized currents. hERG current was measured using a 2 s step protocol: depolarization to 20 mV (2 s), followed by repolarization to −40 mV (2 s), repeated every 15 s (holding potential = −80 mV).

Automated patch-clamp: Peak tail current distribution of hERG stable cell line and the block effect of E-4031 on hERG were analyzed using SyncroPatch 384i APC system (Nanion Technologies, Munich, Germany). At 70–80% confluence, stable hERG cells were harvested with TrypLE (Sigma-Aldrich, Sydney, Australia), washed with cold (4 °C) FBS buffer, and resuspended in cold external solution (140 mM NaCl, 4 mM KCl, 2 mM CaCl_2_, 1 mM MgCl_2_, 5 mM glucose, 10 mM HEPES; pH 7.4) to 1.0–1.5 × 10^6^ cells/mL. Whole-cell recordings followed Nanion’s protocol: a 384-well recording chamber (NPC-384, Nanion Technologies) with 1 patch aperture/well was filled with internal fluid (10 mM NaCl, 10 mM KCl, 110 mM KF, 10 mM HEPES, 10 mM EGTA; pH 7.2) via perfusion and divalent-free external solution (140 mM NaCl, 4 mM KCl, 5 mM glucose, 10 mM HEPES; pH 7.4) by 384-multichannel pipette tip. Cells stored at 10 °C (200 rpm shake speed) were then added to the chip. Fifty percent of the divalent-free solution was then exchanged with Ca^2+^-containing seal enhancer solution (130 mM NaCl, 4 mM KCl, 10 mM CaCl_2_, 1 mM MgCl_2_, 5 mM glucose, 10 mM HEPES; pH 7.4) to enhance seal resistance, and cells were washed twice with external solution. After establishing whole-cell configuration and recording baseline currents, compounds were added. The voltage-clamp protocol matched the manual patch-clamp, and peak tail current at −40 mV was defined as hERG current amplitude. Valid whole-cell recordings had to meet the following criteria: seal resistance > 400 MΩ, series (access) resistance <15 MΩ, cell capacitance 5–50 pF.

Data analysis: All the data were presented as mean ± standard deviation (s.d.) unless otherwise specified. All statistical analyses were performed using Prism software (GraphPad Prism version 9.4.1; www.graphpad.com). Compound effect was calculated using the following formula: %block = (control − drug)/control ∗ 100. IC_50_ values were calculated by fitting dose–responses to the four-parameter Hill equation using GraphPad Prism with constrained bottom at 0 and top at 1.

## 5. Conclusions

By combining stable hERG expression with APC efficiency, this platform facilitates early detection of hERG-related cardiac safety risks, guiding drug candidate optimization to reduce cardiotoxicity and improve clinical trial success rates—ultimately supporting safer, more effective medication development. Future work may explore its application in screening diverse chemical libraries and investigating mechanisms of hERG channel modulation.

## Figures and Tables

**Table 1:** Current distributions of stable HEK293T-hERG cells detected by APC system.

Amplitude (nA)	<0.05	0.05–0.1	0.1–0.2	0.2–0.4	0.4–0.6	0.6–0.8	0.8–1	>1
Number	14	14	30	51	45	30	16	16
Ratio (%)	6.5	6.5	13.9	23.6	20.8	13.9	7.4	7.4

## Data Availability

All data supporting the findings of this study are available within the article.

## Supplementary Materials

The following supporting information can be downloaded at https://www.mdpi.com/article/10.3390/ijms27083701/s1.
