# Supplementary material for: Establishment and Characterization of a Stable hERG Cell Line for High-Throughput Drug Cardiac Safety Screening
Source: Int J Mol Sci. 2026 Apr 21;27(8):3701. doi: 10.3390/ijms27083701 (PMC13116348; doi:10.3390/ijms27083701)
Supplement: Supplementary file 1 [file ijms-27-03701-s001.zip › ijms-4226780-supplementary.pdf]

Communiaction

Establishment and Characterization of a Stable hERG Cell Line for High-Throughput Drug Cardiac Safety Screening

Hailin Lu [1], Qingqing Guo [2], Qinling Qiu [3] and Jiying Hu [1] *

[1] High-throughput Screening Center, Shenzhen Bay Laboratory, Shenzhen 518132 China; luhl@szbl.ac.cn
[2] Biomedical Research Core Facility, Shenzhen Bay Laboratory, Shenzhen 518132 China; guoqq@szbl.ac.cn
[3] Institute of Molecular Physiology, Shenzhen Bay Laboratory, Shenzhen 518132 China; qiuql@szbl.ac.cn
* Correspondence: address. Tel: +86-18086481821; E-mail: hujy@szbl.ac.cn

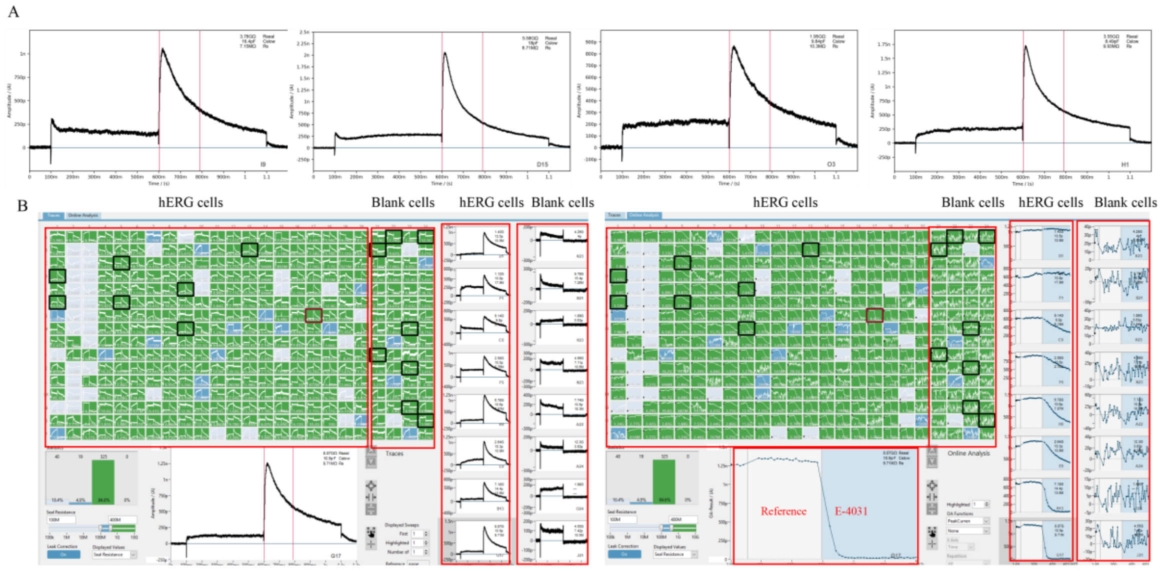

Figure S1. Representative hERG channel current recordings and pharmacological analysis of E-4031 inhibition via automated patch-clamp (APC) platform. (A) Representative hERG channel current traces recorded by the APC platform. (B) Left panel: Screenshot of whole-plate current recordings, where columns 1-20 are hERG-expressing cells and columns 21-24 are blank cells; Right panel: Quantitative online analysis of peak tail currents. The time period with a white background represents the reference treatment, and the time period with a blue background represents the E-4031 treatment.
